# Assessing Age-Related Etiologic Heterogeneity in the Onset of Islet Autoimmunity

**DOI:** 10.1155/2015/708289

**Published:** 2015-03-25

**Authors:** Brittni N. Frederiksen, Miranda Kroehl, Anna Barón, Molly M. Lamb, Tessa L. Crume, Marci K. Sontag, Marian Rewers, Jill M. Norris

**Affiliations:** ^1^Department of Epidemiology, Colorado School of Public Health, University of Colorado Denver, 13001 E. 17th Place, Box B119, Aurora, CO 80045, USA; ^2^Department of Biostatistics and Informatics, Colorado School of Public Health, University of Colorado Denver, 13001 E. 17th Place, Box B119, Aurora, CO 80045, USA; ^3^Barbara Davis Center for Childhood Diabetes, University of Colorado Denver, 1775 Aurora Ct., Aurora, CO 80045, USA

## Abstract

Type 1 diabetes (T1D), a chronic autoimmune disease, is often preceded by a preclinical phase of islet autoimmunity (IA) where the insulin-producing beta cells of the pancreas are destroyed and circulating autoantibodies can be detected. The goal of this study was to demonstrate methods for identifying exposures that differentially influence the disease process at certain ages by assessing age-related heterogeneity. The Diabetes Autoimmunity Study in the Young (DAISY) has followed 2,547 children at increased genetic risk for T1D from birth since 1993 in Denver, Colorado, 188 of whom developed IA. Using the DAISY population, we evaluated putative determinants of IA, including non-Hispanic white (NHW) ethnicity, maternal age at birth, and erythrocyte membrane n-3 fatty acid (FA) levels, for age-related heterogeneity. A supremum test, weighted Schoenfeld residuals, and restricted cubic splines were used to assess nonproportional hazards, that is, an age-related association of the exposure with IA risk. NHW ethnicity, maternal age, and erythrocyte membrane n-3 FA levels demonstrated a significant age-related association with IA risk. Assessing heterogeneity in disease etiology enables researchers to identify associations that may lead to better understanding of complex chronic diseases.

## 1. Introduction

Type 1 diabetes (T1D) results from the destruction of the insulin-producing pancreatic beta cells. The incidence of T1D is increasing at an annual rate of about 3% worldwide [1]. The most rapid increase has been in children younger than 5 years old [1–4].

T1D is preceded by a preclinical phase of islet autoimmunity (IA) where the body produces antibodies (IAA, GAD_65_, or IA-2) against the insulin-producing beta cells of the pancreas, which can be detected as early as 6 months of age [5]. There appears to be two peaks in IA incidence at approximately 1-2 years of age and in adolescence, with distinct characteristics at each peak [5].

IA and T1D development may be subject to age-related etiologic heterogeneity, where exposures influence the disease process more strongly at certain ages. T1D development is more likely to occur earlier in life for those with disease-associated HLA genotypes and a parental history of T1D [6–12]. A recent study found differences in serum metabolite profiles relative to age; an association between lower methionine levels and presence of diabetes autoantibodies in younger onset (≤2 years) but not older onset (≥8 years) autoimmunity was described [13]. Additionally, Virtanen et al. found early introduction of wheat, rye, oats and/or barley cereals, and egg was associated with increased IA risk, but only during the first 3 years of life, suggesting an age-related association [14].

Assessment of age-related heterogeneity allows understanding of* if* and* when* exposures play a role in the disease process. Valuable associations may be overlooked if they are averaged across ages and not evaluated for heterogeneity. Knowing when exposures play a role in the disease process can guide treatment and prevention efforts by creating more accurate risk prediction models and informing the design of targeted interventions.

Prospective cohorts of children at increased T1D risk are often followed from birth to IA and T1D development. Time-to-event analyses, frequently implemented using Cox proportional hazards (PH) regression, are utilized to identify risk factors. A Cox PH model assumes the hazard ratio (HR) is constant over time, meaning the association of a covariate is the same at all time points. If age-related heterogeneity is present for a given variable, the association of that variable changes over time (i.e., age) and the PH assumption is not valid. Therefore, age-related heterogeneity can be assessed by evaluating the PH assumption. We demonstrate the use of three methods for testing and modeling non-PH: a supremum test, evaluation of weighted Schoenfeld residuals, and restricted cubic splines.

### 1.1. Supremum Test

The supremum test, a regression diagnostic for PH models, plots the path of the observed cumulative sum of martingale residuals for a covariate against time [15]. Rather than a test statistic, it produces a* P* value which represents the percentage of 1000 simulated paths embodying the PH assumption whose supremum (or largest) values exceed the supremum of the observed path for the covariate of interest [15]. Higher* P* values (ideally much greater than 0.05) are a stronger indication that the PH assumption holds, suggesting the supremum of the observed path is substantially smaller than a large proportion of the suprema of the 1000 simulated paths that actually follow the PH assumption for that covariate [15]. The test is implemented in SAS PROC PHREG.

### 1.2. Weighted Schoenfeld Residuals

Weighted Schoenfeld residuals can be plotted as another PH regression diagnostic as described by Grambsch and Therneau [16]. In the R package using the cox.zph function of the* survival* library, these residuals produced separately for each covariate for each individual are visualized through scatterplot smoothing. This effectively shows how the regression coefficient, *β*(*t*), varies with time [16]. If the assumption of PH is satisfied, the residuals will be independent of time; thus, a non-zero slope indicates a violation of the PH assumption.

### 1.3. Restricted Cubic Splines

Restricted cubic splines (RCS) are piecewise polynomials smoothly joined at* k* knot values that can also be used to identify and model non-PH [17, 18]. RCS provide a statistical test as well as a visual assessment of the HR as a function of time and allow for flexible modeling of the HR without a specific functional form, for example, linear or quadratic. The number of knots selected for the splines is chosen based on Akaike information criterion (AIC), where a lower value indicates better fit. The SAS RCS macro, designed to assess PH for fixed covariates, first tests whether the covariate of interest is associated with the event. If the covariate is associated with the event, one can then test whether the association is nonconstant with time (indicating a violation of the PH assumption) and, if so, whether the relationship between the HR and time is linear or not [17, 18].

### 1.4. Diabetes Autoimmunity Study in the Young (DAISY)

DAISY has prospectively collected 20 years of data from birth on children at increased genetic risk for T1D. DAISY data can be used to study prospective exposures across childhood and assess whether the risk associated with determinants of IA and T1D differs by age when the child develops IA and/or T1D. T1D has a complex etiology with numerous identified factors that either increase or decrease disease risk. The goal of this study was to demonstrate the aforementioned three methods of identifying exposures that influence the disease process at varying ages by assessing age-related heterogeneity (or lack of PH) of putative IA determinants, including non-Hispanic white (NHW) ethnicity, maternal age at birth, and erythrocyte membrane n-3 fatty acid (FA) levels. We were interested in assessing age-related heterogeneity of IA because if risk factors are shown to have associations that vary by age, then age-appropriate interventions can be designed to prevent or slow the development of IA and, ultimately, T1D.

## 2. Materials and Methods

### 2.1. Study Population

DAISY recruited two groups of children between 1993 and 2004, who are at increased T1D risk and followed prospectively for IA and T1D development. One group consists of first-degree relatives of patients with T1D, identified and recruited between birth and age 8, mainly through the Barbara Davis Center for Childhood Diabetes. The second group consists of infants born at St. Joseph's Hospital in Denver, CO, whose umbilical cord blood was screened for diabetes-susceptibility HLA-DR, DQ genotypes and recruited if they had these genotypes [19, 20]. Details of the newborn screening, sibling and offspring recruitment, and follow-up of both cohorts have been published previously [21]. Cord blood or the first available blood sample (depending on enrollment group) was sent to Roche Molecular Systems, Inc., Alameda, CA, for PCR-based HLA-DR, DQ typing. All study protocols were approved by the Colorado Multiple Institutional Review Board, and informed consent was given by parents of all participating children.

**Figure 1:**
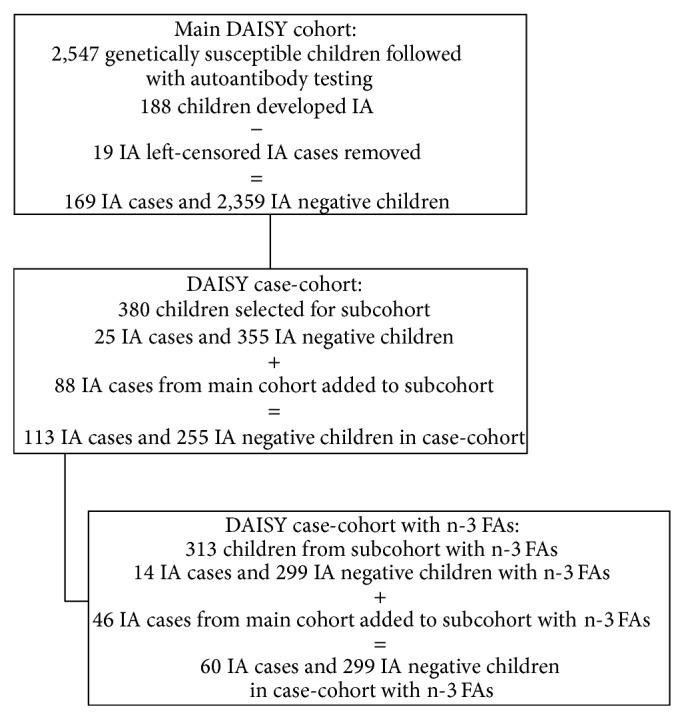
Flow chart illustrating the formation of the cohorts for the investigation of age-related heterogeneity.

The DAISY cohort is composed of 2,547 children, of whom 188 have developed IA. Nineteen IA cases were positive for autoantibodies on their first clinic visits; these left-censored cases were removed from the analysis. We examined NHW ethnicity and maternal age at birth as fixed covariates for age-related heterogeneity in this cohort. We also assessed the time-varying covariate, erythrocyte membrane n-3 FA levels, for age-related heterogeneity. As described previously [22], erythrocyte membrane n-3 FA levels were investigated in a case-cohort design, for which a representative sample of 380 children (i.e. subcohort) was selected from the main DAISY cohort using stratified sampling based on HLA-DR genotype and family history of T1D; 313 of these children had erythrocyte n-3 FA measurements. Erythrocyte n-3 FA levels were measured at 9, 15, and 24 months of age and annually thereafter. The median age at which n-3 FA levels were determined was 6.9 years (IQR: 5.2 years). During follow-up, 14 children with erythrocyte membrane n-3 FA levels developed IA within the subcohort. We supplemented these with 46 children who developed IA outside of the subcohort to complete our case-cohort study population. Therefore, 60 children with IA and 299 IA negative children were included in the analysis of erythrocyte membrane n-3 FA levels (see Figure 1 for flow-chart illustrating the formation of the analysis cohorts).

### 2.2. Measurement of Erythrocyte Membrane n-3 FA Levels

Erythrocytes from blood samples were separated within 30 minutes of blood draw, flash-frozen in liquid nitrogen, and stored at −70°C. Lipids were extracted from erythrocytes following the method developed by Bligh and Dyer [23] and stored at −20°C in sealed cryotubes following flushing with nitrogen gas. The FAs present in the lipid isolates were methylated using the base-catalyzed procedures by Maxwell and Marmer [24] in preparation for analysis by gas chromatography (Hewlett-Packard 6890; Agilent, Santa Clara, CA, USA) with mass spectral detection (Hewlett-Packard 5973; Agilent). The samples, separated across a CP-WAX column (25 m × 0.25 mm internal diameter, 0.2-*µ*m film; Varian, Palo Alto, CA, USA), were identified by comparing the retention times and* m/z* of selected ions from analytes in the samples with those of authentic standards (NuCheckPrep; Elysian, MN, USA; Supelco; St. Louis, MO, USA). Quantification was determined against five-point standard curves and FA percentage is reported as a percent of total lipids (g of FA/100 g erythrocyte lipid). The following n-3 FAs were measured in the membranes and combined to estimate total n-3 FAs in the membrane (as a percent of total lipids): *α*-linolenic acid (ALA) (18:3n*-*3), eicosapentaenoic acid (EPA) (20:5n-3), docosahexaenoic acid (DHA) (22:6n-3), and docosapentaenoic acid (DPA) (22:5n-3).

### 2.3. Measurement of Autoantibodies

Autoantibodies were tested at 9, 15, and 24 months, or at their first visit (if enrolled after the first year of life), and annually thereafter. Radioimmunoassays measured serum autoantibodies to insulin, glutamic acid decarboxylase (GAD)_65_, and insulinoma antigen (IA-2) (also known as BDC512), as previously described [25–27], with confirmation of all positive and a subset of negative results. Cut-off for positivity was established at the 99th percentile of healthy controls. Children testing autoantibody positive were put on an accelerated testing schedule of every 3–6 months. IA cases were defined as children positive for at least one autoantibody (IAA, GAD_65_, IA-2) on ≥2 consecutive visits within 6 months with the end of follow-up in children who developed IA defined as the first of the antibody positive visits and the last negative sample in children who did not develop IA (median duration of follow-up was 8.1 years (IQR: 6.3 years)).

### 2.4. Statistical Analyses

#### 2.4.1. Assessment of the PH Assumption

We assessed violation of the PH assumption using three different methods: the supremum test, weighted Schoenfeld residuals, and restricted cubic splines. The supremum test was performed in SAS version 9.3 (SAS Institute, Cary, NC) using the ASSESS statement in the PHREG procedure. Weighted Schoenfeld residuals were plotted using the cox.zph function in R 2.15.2 [28]. A significant supremum test (*P *< 0.30) or a non-zero slope for the loess smoothed curve of the weighted Schoenfeld residuals indicated a violation of PH [16]. Using the weighted Schoenfeld residuals, a global test of PH was assessed first; if the global test* P* value was not large (*P* < 0.30), the individual covariate tests of PH were used to identify the source(s) of the non-PH. Restricted cubic splines were modeled using the RCS macro in SAS [17, 18]. Due to a limited number of events, the number of knots for the RCS was selected to be 3, placed at the 5th, 50th, and 95th percentiles of age of the IA cases; this minimizes the number of coefficients to fit the RCS models [17, 18]. The RCS macro provides the three statistical tests described above which should be performed hierarchically. The first test has 3 df for a 3-knot spline model and tests whether the covariate of interest is associated with the event. If the null hypothesis is rejected (*P* < 0.05), the second statistical test with 2 df for a 3-knot spline model can be performed to determine whether the association is nonconstant with time (*P* < 0.05 indicating a violation of PH). Finally, if the null hypothesis is rejected for both the first and second statistical tests, the third statistical test with 1 df for a 3-knot spline model can be performed to determine whether the relationship between the HR and time is linear (*P* < 0.05 indicates nonlinearity) [17, 18].

#### 2.4.2. Fixed Covariates

The three methods (supremum test, weighted Schoenfeld residuals, and RCS) were evaluated for the fixed covariates: NHW ethnicity and maternal age at birth. Statistical analyses were conducted using SAS software, Version 9.3 of the SAS System for Windows. Copyright © 2002–2010 SAS Institute Inc. SAS and all other SAS Institute Inc. product or service names are registered trademarks or trademarks of SAS Institute Inc., Cary, NC, USA, except for the weighted Schoenfeld residuals, which were generated and plotted in R [29].

#### 2.4.3. Time-Varying Covariates

Existing tools are limited with regard to examination of PH with time-varying covariates. The supremum test for violation of the PH assumption can theoretically accommodate time-varying covariates but requires higher dimensional plots for time-varying covariates and is not implemented in standard statistical software. The cox.zph function used to plot the weighted Schoenfeld residuals is valid for time-varying covariates; however, the software assumes the variance of the time-varying covariate is constant over time [16]. If this assumption is violated, results from the weighted Schoenfeld residual tests are not reliable. Motivated by these limitations in assessing PH with time-varying covariates, Kroehl et al. (unpublished) recently adapted RCS for use with time-varying covariates and evaluated their performance in identifying and modeling a nonconstant HR. Using this approach, non-PH was investigated for erythrocyte membrane n-3 FA levels.

## 3. Results

Table 1 describes the DAISY children by IA status. Of 188 IA-positive children in DAISY, 19 were excluded from analyses of IA because they tested autoantibody positive on their first study visit (i.e., left-censored). Median age at first IA-positive visit was 6.0 years and 9.0 years at last follow-up visit in those without IA. Children who developed IA were more likely to have the HLA-DR3/4, DQB1^*^0302 genotype and have a first-degree relative with T1D compared to children who did not develop IA (Table 1).  Table 2 shows the characteristics of the DAISY subcohort by IA status in which the median age at first IA-positive visit was 5.1 years and 8.6 years at last follow-up visit in those without IA.

### 3.1. Fixed Covariates

**Figure 2:**
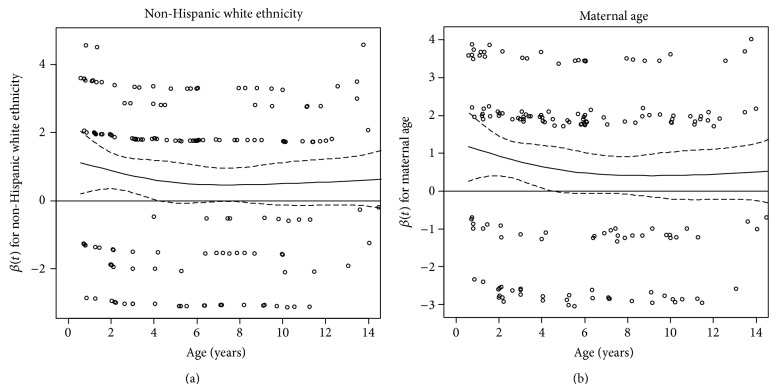
The weighted Schoenfeld residual plots are displayed for non-Hispanic white ethnicity (NHW) (a) and a 5-year difference in maternal age (b) in the prospective DAISY cohort. The* x*-axis represents age in years and the* y*-axis represents the coefficient estimate for non-Hispanic white ethnicity in (a) and coefficient for maternal age in (b). The dots represent the residuals for each individual. The solid line is a smoothing-spline fit to the plot, with the dashed lines representing the 95% confidence interval. The global PH test* P* values based on the Schoenfeld residuals are 0.02 and 0.01 for NHW ethnicity and a 5-year difference in maternal age, respectively, indicating a violation of the PH assumption.

**Figure 3:**
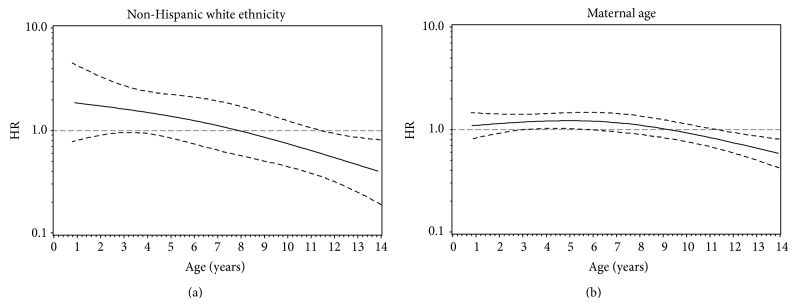
A restricted cubic spline model was used to estimate the hazard ratio as a function of age. The restricted cubic spline for non-Hispanic white ethnicity exhibits an increased risk of islet autoimmunity (IA) early on that then becomes protective in the older ages (a). The restricted cubic spline for a 5-year difference in maternal age exhibits a slightly elevated risk of IA in early childhood that becomes protective in adolescence (b). The* x*-axis represents age in years and the* y*-axis represents the hazard ratio on the log scale. The solid line represents the hazard ratio and the dashed lines represent the pointwise 95% confidence bands.

We first assessed age-related heterogeneity of two fixed covariates: NHW ethnicity and maternal age at birth. The supremum test* P *value was 0.01 for NHW ethnicity adjusting for HLA-DR3/4, DQB1^*^0302 genotype and first-degree relative status, indicating a violation of PH (Table 3). The weighted Schoenfeld residuals had a global PH test *P* = 0.02 and an individual PH test *P* = 0.01, indicating a violation of PH (Figure 2(a)). We modeled the RCS to evaluate the HR as a function of time. NHW ethnicity showed an overall significant association with IA risk adjusting for HLA-DR3/4, DQB1^*^0302 genotype and first-degree relative status (Association *P* = 0.03) (Table 3). Based on rejection of the null hypothesis, the nonconstant association was tested, producing a* P *= 0.01, indicating non-PH (Table 3). Finally, a nonlinear association was tested based on rejection of the null hypothesis for the nonconstant association, which was not significant (Nonlinear *P* = 0.62), indicating a linear decrease in IA risk associated with NHW ethnicity over time (Table 3). The restricted cubic spline demonstrated an elevated risk in early childhood (age 2 HR: 1.74, 95% CI: 0.90, 3.36) diminishing with increasing age (age 11 HR: 0.64, 95% CI: 0.38, 1.06) (Figure 3(a)).

The supremum test* P* value for maternal age was 0.01 adjusting for HLA-DR3/4, DQB1^*^0302 genotype and first-degree relative status, indicating a violation of PH (Table 3). The weighted Schoenfeld residuals for maternal age also had a significant global PH test *P* = 0.01 and an individual PH test *P* = 0.003, another indication of a PH violation (Figure 2(b)). The modeled RCS resulted in a significant overall association of maternal age with IA risk adjusting for HLA-DR3/4, DQB1^*^0302 genotype and first-degree relative status (Association *P* = 0.003) (Table 3). Based on rejection of this null hypothesis, the nonconstant association of maternal age was tested with a resulting *P* = 0.001, demonstrating age-related heterogeneity (Table 3). Finally, based on rejection of the null hypothesis for the nonconstant association test, a nonlinear association was tested. In contrast with the restricted cubic spline result for NHW ethnicity, the nonconstant association of maternal age for a five-year difference was also nonlinear (Nonlinear *P* = 0.03) with greater maternal age at birth associated with increased risk in early childhood (age 2 HR: 1.14, 95% CI: 0.92, 1.42), which became protective later in adolescence (age 11 HR: 0.83, 95% CI: 0.68, 1.02) (Figure 3(b)).

### 3.2. Time-Varying Covariates

**Figure 4:**
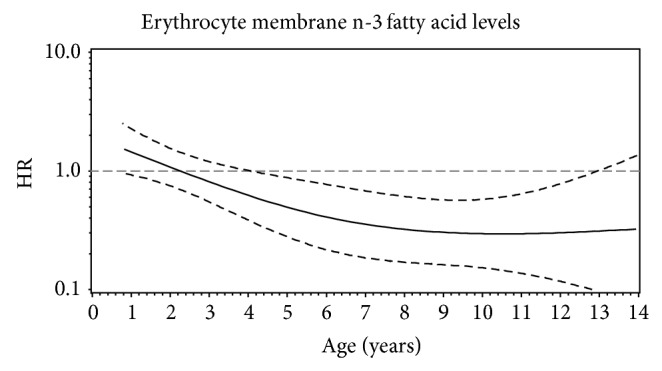
The restricted cubic spline function for erythrocyte membrane n-3 fatty acid levels and islet autoimmunity development in the prospective DAISY cohort displays a linear decrease in risk across childhood. The* x*-axis represents age in years and the* y*-axis represents the hazard ratio on the log scale. The solid line represents the hazard ratio and the dashed lines represent the pointwise 95% confidence bands.

We then assessed age-related heterogeneity for the time-varying covariate, erythrocyte membrane n-3 FA levels, by fitting a restricted cubic spline model (Figure 4). Erythrocyte membrane n-3 FA levels had an overall significant association with IA risk (Association *P* = 0.001) adjusting for HLA-DR3/4, DQB1^*^0302 genotype and first-degree relative status, previously established in DAISY by Norris et al. [21]. With rejection of this null hypothesis, the nonconstant association of erythrocyte membrane n-3 FA levels was tested and resulted in a *P* = 0.001, indicating non-PH (Figure 4). Finally, a nonlinear association was tested based on rejection of the null hypothesis for the nonconstant association, which was not significant (*P* = 0.17), indicating a linear decrease in IA risk associated with erythrocyte membrane n-3 FA levels over time (age 2 HR: 1.08, 95% CI: 0.75, 1.56 and age 11 HR: 0.30, 95% CI: 0.14, 0.64) (Table 3 and Figure 4).

## 4. Discussion

This study elucidates potential challenges in identifying triggers of T1D, which are associations that change with age. We demonstrate three age-related associations of putative IA determinants that would be masked if averaged across age and not evaluated for heterogeneity. Knowing when exposures play a role in the disease process is important in better understanding complex diseases. RCS aid in visualizing risk over time and provide statistical tests to determine whether risk is nonconstant with respect to time or age.

The assumption of PH is central to Cox PH analysis, and if violated, inferences made from an analysis may be incorrect. For example, the association of an age-sensitive risk factor could be missed if the association, averaged over time, is determined to be nonsignificant. All three of the variables presented here violated the PH assumption, indicating it may be somewhat common for risk factors to be subject to age-related heterogeneity. It is important to recognize even if a covariate does not have a statistically significant* P* value for the nonconstant association when assessing non-PH with RCS, marginal associations may be clinically interesting or meaningful. RCS also provide HRs and 95% CIs for any time point or age of interest. Attributable risks can then be calculated to determine whether the number of cases attributed to the disease at a certain age is meaningfully different from other ages [30–32].

We were interested in assessing age-related heterogeneity of IA, because if IA risk factors can be identified, preventive efforts may be designed to slow or even prevent progression to T1D. NHW ethnicity, maternal age, and erythrocyte membrane n-3 FA levels that demonstrated significant age-related heterogeneity with statistically significant* P* values for the nonconstant association have not been previously shown to demonstrate etiologic heterogeneity in T1D.

The linear decrease in IA risk associated with NHW ethnicity may reflect the fact that the age of IA development in DAISY is younger in NHW children compared to other racial/ethnic groups (mean: 6.0 years in NHW children versus 7.9 years in children of other racial/ethnic groups). Another study in DAISY found that children who developed IA after age 7 years were more likely than those who developed IA before age 7 years to be ethnic minorities [33]. A study using the Colorado IDDM Registry found that, on average, NHW children developed T1D six months earlier than Hispanic children (mean: 9.5 years versus 10.0 years) [34]. This may also reflect a higher genetic load of non-HLA T1D genotypes in NHW children resulting in a younger disease onset in NHW children. Children of other racial/ethnic groups may develop T1D later, reflecting a different disease that may initially appear to be type 2 diabetes but is, in fact, T1D.

Our finding with maternal age may be interesting for hypothesis generation. The slight increased IA risk associated with increased maternal age at birth in younger children may be due to a number of perinatal exposures. Older mothers may be more likely to have complicated pregnancies that result in cesarean or complicated vaginal deliveries (i.e., breech or use of forceps or vacuum), increasing their child's risk of IA and T1D development [35–38]. We have also observed that older mothers are more likely to delay the introduction of solid foods, including cereals, which has been associated with an increased risk of IA and T1D [37, 39]. Another explanation may be that older mothers have more accumulated exposures throughout their lifetime, which may influence biological programming in the child [35, 40]. These early risk factors for T1D may only exert influence in the early years and once these susceptible children develop IA, maternal age may no longer be associated with increased IA risk. Younger mothers are more likely to have lower SES and educational attainment, which could start to have an influence as children get older by resulting in fewer opportunities for extracurricular physical activity or healthy food choices for their children in adolescence [41], possibly increasing IA risk. It is also possible that NHW ethnicity and maternal age are measuring similar things, as mothers of NHW children are significantly older than mothers of children of other racial/ethnic groups in our population (mean: 30.6 years versus 27.8 years, resp.).

The inverse association between n-3 FA levels and IA risk after age 4.25 may provide an important age for the efficacy of n-3 FAs and future n-3 FA intervention studies. In our population, increased n-3 FA levels were not significantly associated with IA risk before age 4.25 years. It is possible that only a small portion of young children consume fatty fish, the primary dietary source of n-3 FA, resulting in limited variability in n-3 FA levels and making it difficult to see an effect of n-3 FA levels in early childhood. The variability in n-3 FA intake in children younger than 4.25 years in the DAISY population is significantly lower than the variability in n-3 FA intake in children 4.25 years and older (mean ± SD: 1.09 ± 0.49 versus 1.19 ± 0.57, resp.).

Assessing age-related heterogeneity is an important step in understanding etiologic heterogeneity of complex diseases and ensuring key associations are not overlooked. We encourage researchers using Cox PH regression analyses to assess covariates for violation of the PH assumption when using them in a Cox PH model. As a preliminary step preceding assessment of PH, continuous covariates should be examined for correct functional form with all important covariates and confounders included in the model [42]. If the variable is not linearly associated with the HR, a transformation may be appropriate (such as a log transform) prior to assessing for PH. Martingale residuals are a common diagnostic tool for evaluating functional form. It should also be recognized that missing covariates can also erroneously induce nonproportionality [42].

Regarding the three methods for testing the PH assumption presented in this paper, we suggest the supremum test or the weighted Schoenfeld residuals for diagnostic testing of the PH assumption to determine if a fixed covariate can be used in a Cox PH model. However, if one is interested in examining the nature of age-related heterogeneity or evaluating the PH assumption with a time-varying covariate, we recommend modeling RCS and performing the hierarchical testing of the PH assumption by first determining whether the covariate of interest is associated with the event. If the null hypothesis is rejected and the covariate is associated with the event, a statistical test can be performed to determine whether the association is nonconstant with time, violating the assumption of PH. Using this approach, DAISY recently detected a single nucleotide polymorphism, rs10517086, that demonstrated age-related heterogeneity with IA risk, with increased risk before 2 years of age (age 2 HR: 1.67, 95% CI: 1.08, 2.56), but not older ages (age 4 HR: 0.84, 95% CI: 0.43, 1.62) [43].

## 5. Conclusions

RCS are a powerful way of visualizing the true form of an exposure with time with the ability to test whether this association exhibits a nonconstant or nonlinear association. They have the added advantage of being applicable to time-varying exposures. This method may aid in identifying or confirming environmental determinants that play a role in the etiology of T1D.

## Figures and Tables

**Table 1:** Characteristics of main DAISY cohort by IA status (*n* = 2,528).

Characteristic	Number (%)		*P* value
	Developed IA (*n* = 169)	Did not develop IA (*n* = 2,359)	
Age, median (IQR), y	6.0 (6.6)^1^	9.0 (10.9)^2^	<0.0001
HLA-DR3/4, DQB1^*^0302 genotype	63 (37%)	435 (18%)	<0.0001
First-degree relative with T1D	100 (59%)	1019 (43%)	<0.0001
Female	88 (52%)	1126 (48%)	0.28
Race/ethnicity, non-Hispanic white	132 (78%)	1690 (72%)	0.10
Maternal age, mean (SD), y	30.3 (5.8)	29.8 (5.7)	0.25

DAISY, Diabetes Autoimmunity Study in the Young; HLA, human leukocyte antigen; IA, islet autoimmunity; T1D, type 1 diabetes.

^1^Age at first IA positive visit.

^2^Age at last follow-up.

**Table 2:** Characteristics of DAISY subcohort by IA status (*n* = 359).

Characteristic	Number (%)		*P* value
	Developed IA (*n* = 60)	Did not develop IA (*n* = 299)	
Age, median (IQR), y	5.1 (6.3)^1^	8.6 (6.3)^2^	<0.0001
HLA-DR3/4, DQB1^*^0302 genotype	21 (35%)	83 (28%)	0.26
First-degree relative with T1D	31 (52%)	118 (39%)	0.08
Female	35 (58%)	148 (50%)	0.21
Race/ethnicity, non-Hispanic white	47 (78%)	222 (74%)	0.51
Maternal age, mean (SD), y	30.5 (5.5)	30.0 (5.4)	0.47

DAISY, Diabetes Autoimmunity Study in the Young; HLA, human leukocyte antigen; IA, islet autoimmunity; T1D, type 1 diabetes.

^1^Age at first IA positive visit.

^2^Age at last follow-up.

**Table 3:** Assessment of the proportional hazards assumption in DAISY cohort.

Characteristic	Supremum test *P*	Schoenfeld residuals global *P*	Schoenfeld residuals individual covariate *P*	Restricted cubic splines association *P*	Restricted cubic splines nonconstant *P*	Restricted cubic splines nonlinear *P*
Non-Hispanic white ethnicity^1^	0.01	0.02	0.01^2^	0.03^3^	0.01^3^	0.62^3^
Maternal age^1^	0.01	0.01	0.003^4^	0.003^5^	0.001^5^	0.03^5^
Erythrocyte membrane *n*-3 fatty acid levels^1^	N/A	N/A	N/A	0.001^6^	0.001^6^	0.17

DAISY, Diabetes Autoimmunity Study in the Young; HLA, human leukocyte antigen; T1D, type 1 diabetes.

N/A: statistical tests were not performed based on the inability to reject the null hypothesis of the prior test in the hierarchical structure.

^1^Adjusted for HLA-DR3/4, DQB1^*^0302 genotype and first-degree relative with T1D.

^2^ Figure 2(a).

^3^ Figure 3(a).

^4^ Figure 2(b).

^5^ Figure 3(b).

^6^ Figure 4.
